# Presence of the Endocannabinoid System in the Inferior Pulvinar of the Vervet Monkey

**DOI:** 10.3390/brainsci11060770

**Published:** 2021-06-10

**Authors:** Catarina Micaelo-Fernandes, Joseph Bouskila, Jean-François Bouchard, Maurice Ptito

**Affiliations:** 1 École d’Optométrie, Université de Montréal, Montreal, QC H3T 1P1, Canada; catarina.fernandes@umontreal.ca (C.M.-F.); joseph.bouskila@umontreal.ca (J.B.); jean-francois.bouchard@umontreal.ca (J.-F.B.); 2 Department of Neuroscience, Copenhagen University, 2200 Copenhagen, Denmark; 3 Department of Neurology and Neurosurgery, Montreal Neurological Institute, McGill University, Montreal, QC H3A 2B4, Canada

**Keywords:** vervet monkeys, pulvinar, endocannabinoids, CB1R, FAAH, NAPE-PLD, vision

## Abstract

The expression of the endocannabinoid (eCB) system, including cannabinoid receptor type 1 (CB1R) and the cannabinoid synthesizing (NAPE-PLD) and degrading (FAAH) enzymes, has been well-characterized in the retina of rodents and monkeys. More recently, the presence of CB1R was localized throughout the dorsal lateral geniculate nucleus of the thalamus of vervet monkeys. Given that the retina projects also to the pulvinar either via a direct projection or via the superior colliculus, it was reasonable to assume that this system would be present therein. The visual pulvinar, namely the inferior pulvinar (PI) region, was delineated with calbindin immunohistochemical staining. Using Western blots and immunofluorescence, we demonstrated that CB1R, NAPE-PLD and FAAH are expressed in the PI of the vervet monkey. Throughout the PI, CB1R was mainly colocalized with VGLUT2-positive axon terminals in the vicinity of calbindin and parvalbumin-positive neurons. NAPE-PLD and FAAH rather colocalized with calbindin over the somatodendritic compartment of PI neurons. Our results suggest that visual information coming from the retina and entering the PI is modulated by the eCB system on its way to the dorsal visual stream. These results provide insights for understanding the role of eCBs in the modulation of visual thalamic inputs and, hence, visual perception.

## 1. Introduction

Classically, the pulvinar has been ascribed several roles in the processing of visual information including spatial attention [1], visual salience [2], complex motion [3] and even the synchronization of neural activity across large cortical networks through cortico-thalamocortical loops [4]. However, these functions only roughly depict the pulvinar as a highly specialized and well-developed structure in primates as its large number of different nuclei subsystems individually contribute to visual processing.

A clearer picture of the pulvinar’s anatomy and connectivity has emerged with the use of several histological markers and tracers. From a coarse separation into three main subdivisions—the medial (PM), the lateral (PL) and the inferior (PI) pulvinar—various nuclei can now be delineated within the pulvinar complex. The visual pulvinar is constituted by the nuclei integrating the PL and PI subdivisions. The inferior pulvinar subdivision that maintains connections with the dorsal visual stream regroups the posterior (PIp), medial (PIm) and central medial (PIcm) nuclei [5]. Multiple descendant/cortical and ascendant/non cortical inputs converge into the PI to shape its activity and its complexity is greater when the nature of these connections is considered. Sensory information originating in the retina reaches the PI, directly or via the superior colliculus (SC) [6,7], in a unidirectional way. Conversely, the PI establishes bilateral connections with visual cortical areas. These neural signals can be dichotomized into “drivers” and “modulators”, where the former characterize the quality of a specific message conveyed by a reduced number of strong synapses and the latter correspond with the added influence of a multitude of weaker synapses over the intensity of that message [8]. Driving retino-collicular inputs are received by the PIp and the PIcm nuclei and the terminals of neurons projecting from the superficial layers of the superior colliculus into these nuclei can be recognized by the expression of vesicular glutamate transporter 2 (VGLUT2) [9]. In non-human primates, the PIp and the PIcm nuclei project, in turn, to adjoining temporal cortical areas of the middle temporal (MT) complex while they do not seem to project to the MT area itself [10]. Differently, the PIm nucleus receives a direct retinal input and entertains reciprocal projections with the MT area [11]. The nature of this connection was initially thought to be modulatory although recent evidence has challenged this view whereby little glutamatergic parvalbumin-positive neurons (a “driver” phenotype) were identified as the source of this thalamocortical input [12].

One of the key questions still debated concerns the impact of the neuromodulatory systems on the functions of the pulvinar given the wealth of connections it maintains with the visual cortices [13]. Here, we addressed this matter by exploring the distribution of the endocannabinoid (eCB) system in the vervet monkey PI.

Through binding to cannabinoid receptor type 1 (CB1R), a G-protein coupled receptor (GPCR) abundantly expressed in the central nervous system (CNS), endogenous and exogenous cannabinoid ligands produce a vast repertoire of neurobiological effects [14]. Anandamide (arachidonoyl ethanolamide) and 2-arachidonoyl glycerol (2-AG) are two well-characterized endocannabinoid ligands [15]. These compounds are synthetized from membrane lipid precursors, such as arachidonic acid, by *N*-arachidonoyl phosphatidylethanolamine-specific phospholipase D (NAPE-PLD) [16]. Their biosynthesis occurs on demand, being triggered by several types of stimuli including neuronal depolarization, the increase of intracellular calcium concentrations and the activation of metabotropic receptors. Their biological responses are terminated upon degradation by fatty acid amide hydrolase (FAAH) [17]. The eCB system falls under the paradigm of the retrograde synaptic signaling of interneuronal communication whereby eCB ligands produced and released from the postsynaptic neuron act on cannabinoid receptors present in the axon terminals of the presynaptic neuron leading to a reduction of the neurotransmitter release [18,19]. Therefore, a postsynaptic neuron is able to influence its own input and, hence, excitability state in a fine-tuned, efficient and activity-dependent manner. Over time, by modulating the dynamics of neurotransmission locally, retrograde signaling by eCBs can induce short- and long-term modifications in synaptic plasticity, ultimately affecting the development and consolidation of neuronal circuits [18,19,20].

With cumulating evidence gathered from observational and electrophysiological studies suggesting that cannabis intake affects visual processing from as early as the retinal level [21,22], the study of the distribution of the eCB system in the visual system is of utmost value for laying the groundwork for future research. In recent years, our team has demonstrated the presence of CB1R and other eCB systems in the primary visual system (retino-geniculo-striate pathway) of the vervet monkey [23,24,25] and little data, if any, are available for the secondary visual system that includes the pulvinar (retino-pulvino-cortical and retino-colliculo-pulvino-cortical pathways). The goal of this study was therefore to perform a comprehensive neuroanatomical and neurochemical characterization of the eCB system within the inferior pulvinar of the vervet monkey. We hypothesize that the eCB system is expressed and localized in the inferior pulvinar as it is the case for the dorsal lateral geniculate nucleus.

## 2. Materials and Methods

**Animals.** Five young male vervet monkeys (*Chlorocebus sabeus*) were used in this study. The monkeys were born and raised in an enriched environment in the laboratories of the Behavioural Sciences Foundation (BSF; Saint Kitts, West Indies), a facility that is recognized by the Canadian Council on Animal Care (CCAC). Prof. Roberta Palmour (McGill University) donated fresh and fixed brain tissues taken from animals enrolled in a terminal project reviewed and approved by the local Institutional Review Board. They were utilized in accordance with the CCAC requirements for the reduction of animals used for experimental purposes.

**Tissue Preparation.** Coronal brain sections that included the whole pulvinar were obtained following previously published methods [24,26]. Briefly, the animals were sedated with ketamine hydrochloride (10 mg/kg, i.m.) then euthanized with an overdose of sodium pentobarbital (25 mg/kg, i.v.) and perfused transcardially with 0.1 M phosphate buffered saline (PBS, 0.1 M) until complete exsanguination. The brain was then either rapidly fresh frozen for Western blots (WB) or was bathed in a 4% paraformaldehyde solution in PBS for immunohistochemistry. The fixed brain was then stereotaxically blocked, removed from the skull, weighed and the volume determined. The brain was finally cryoprotected in graded sucrose solutions (10%, 15% and 30% sucrose in PBS, 0.1 M) and embedded in Shandon embedding media at −80 °C. The blocks were sliced (40–50 µm) with a cryostat in a serial manner and stored, again according to previously published methods [27].

**Western Blotting.** To test for the presence of the CB1R, NAPE-PLD and FAAH antisera, WBs were performed on fresh vervet pulvinar tissues. For each monkey, the pulvinar from one brain hemisphere was sampled from the PM and PL + PI subdivisions and the tissue was homogenized by hand using a sterile pestle in a RIPA buffer (150 mM NaCl, 20 mM Tris, pH 8.0, 1% NP-40 (USB Corp., Cleveland, OH, USA), 0.5% sodium deoxycholate, 0.1% SDS, 1 mM EDTA) supplemented with a protease-inhibitor mixture (aprotinin 1:1000, leupeptin 1:1000, pepstatin 1:1000 and phenylmethylsulfonyl fluoride 0.2 mg/mL); Roche Applied Science, Laval, QC, Canada). Afterwards, the samples were centrifuged (4 °C, 10 min), the supernatant was extracted and the content was equalized using a Thermo Scientific Pierce BCA Protein Assay Kit (Fisher Scientific, Ottawa, ON, Canada). Ten µg of protein per well was loaded in a 10% sodium dodecyl sulphate (SDS)-polyacrylamide gel and electrophoresed. It was then transferred onto a nitrocellulose membrane filter (BioTrace NTll; Life Sciences, Pall, Pensacola, FL, USA) and washed 3 times for 10 min in TBST (0.15 M NaCl, 25 mM Tris-HCl, 25 mM Tris, 0.5% Tween-20). It was blocked for an hour in 5% skim milk (Selection, Montreal, QC, Canada) in TBST and left to incubate overnight in an IgG primary antibody raised in a rabbit; anti-CB1R, anti-NAPE-PLD and anti-FAAH at a concentration of 1:500 in a blocking solution. On the following day, 6 washes in TBST of 5 min each preceded and followed incubation of the blot in a secondary antibody conjugated to horseradish peroxidase (1:5000; Jackson Immunoresearch, West Grove, PA, USA) in a blocking solution for two hours. An enhanced chemiluminescence (ECL) clarity Western blot substrate was used for protein detection (Bio-Rad, Hercules, CA, USA). The proteins were visualized using ChemiDoc Imaging Software (Bio-Rad, Hercules, CA, USA). For the control condition, the same protocol was run simultaneously as described excepting that the anti-CB1R primary antibody was pre-incubated with its blocking peptide (BP; ab50542; Abcam, Cambridge, UK) in a 1:10 dilution for 1 h prior to incubation with the blot.

**DAB immunohistochemistry.** For the demarcation of nuclei within the PI subdivision, calbindin-D28k (CB) DAB (3,3′-diaminobenzidine) immunostaining was performed in free-floating sections similar to previously published methods [26]. Briefly, brain sections of 40–50 μm that included the pulvinar were cleaned 3 times for 10 min each in a washing solution (0.1 M PBS buffer pH 7.4, 0.03% Triton X-100). The tissue was then protected from non-specific binding in a blocking solution (0.5% triton, 10% either normal donkey serum or normal goat serum in 0.1 M PBS) for 90 min. The tissue was then placed in a primary antibody (Table 1) diluted in a blocking solution and left to incubate overnight at 4 °C. After washing the sections for 10 min once and 5 min twice in a washing solution, the slides were incubated in a secondary antibody (biotinylated goat anti-rabbit diluted 1:200 in a blocking solution) for 2 h. The tissue was then washed 3 times for 10 min and incubated for 1 h in an avidin-biotin-conjugated horseradish peroxidase (Vectastain ABC kit, Burlingame, CA, USA) solution (1:500 in 0.1 M PBS). Another 3 washes of 10 min were performed and the sections were treated with a DAB substrate until the tissue was colored (1 to 10 min). The tissue was then washed again for 3 times of 10 min and the sections were mounted on gelatinized slides and left to dry. They then underwent dehydration in graded ethanol, were cleared in xylene and cover slipped with Permount mounting media (Fisher Scientific; Pittsburgh, PA, USA).

**Immunofluorescence.** Double immunofluorescence labeling was performed on the vervet monkey pulvinar following previously published methods in the retina and dorsal lateral geniculate nucleus [23,24], with minor changes. The tissue was treated the same as in the above DAB protocol for “day one” until primary antibody incubation. When the tissue was ready to be incubated in a primary antibody, it was exposed to two primary antibodies at dilution rates mentioned in Table 1 and incubated overnight. On the second day, the tissue was washed in a washing solution for 3 times for 10 min. The tissue was then incubated in a secondary antibody diluted in a blocking solution (1:200). Afterwards, the slices were stained with SYTOX Orange Nucleic Acid Stain (Molecular Probes, Eugene, OR, USA) diluted in 0.1 M PBS (1:10,000) for 5 min. Finally, the sections were washed 3 times for 10 min in 0.1 M PBS then 1 time for 10 min in 0.1 M PB and then mounted onto gelatinized slides and left to dry for approximately half an hour before coverslipping using a Shandon Immu-mount mounting medium (Thermo Scientific, Pittsburgh, PA, USA).

**Brightfield Microscopy.** The DAB immunohistochemical slides were analyzed under a Leica microscope using a 0.65 × objective and the images were taken using the QCapture software version 3.1.1 (QImaging, Surrey, BC, Canada). All adjustments, such as size, color, brightness and contrast, were performed using ImageJ and Adobe Photoshop (CC; Adobe Systems; San Jose; CA, USA) and subsequently exported into Adobe InDesign (CC; Adobe Systems; San Jose; CA, USA) where the final figure layout was completed.

**Confocal Microscopy.** The fluorescence was detected using an Olympus FV3000 confocal laser-scanning microscope with the cellSens imaging software (Olympus, Richmond Hill, ON, Canada). The images were taken under a 20 × objective at a resolution of 1080 × 1080 pixels. Green and far-red channels were used to detect the signal from the 40 µm slices. The green channel (488 nm) was used to detect the cell markers (CB, PV or VGLUT) and the far-red channels (647 nm) to detect the endocannabinoid markers (CB1R, NAPE-PLD or FAAH).

## 3. Results

### 3.1. Western Blot Analysis

**Figure 1:**
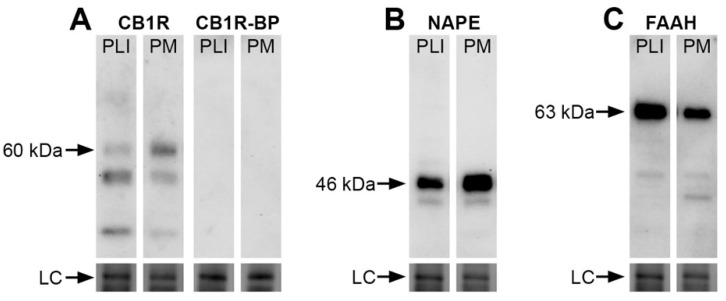
Presence of CB1R, NAPE-PLD or FAAH in the pulvinar of the vervet monkey. A Western blot analysis of the total protein samples of the inferior + lateral (PI + PL) and medial (PM) pulvinar subdivisions for the CB1R antibody (**A**) show the detection of the expected major protein band at 60 kDa. The band was not detected when the antibody was pre-incubated with its corresponding CB1R blocking peptide (BP) at the ratio of 1:10. For the NAPE-PLD antibody, the expected band is seen at 46 kDa (**B**). For the FAAH antibody, the expected band is seen at 63 kDa (**C**). All lanes contained 10 µg of total protein. The loading controls (LC) showing total protein content are displayed at the bottom of the lanes. CB1R: cannabinoid receptor type 1; FAAH: fatty acid amide hydrolase; NAPE-PLD: *N*-acyl phosphatidylethanolamine phospholipase D; PLI: lateral and inferior pulvinar; PM: medial pulvinar.

We investigated the protein expression of three elements of the eCB system by evaluating the total amounts of CB1R as well as the eCB-synthesizing (NAPE-PLD) and degradative (FAAH) enzymes in the monkey pulvinar. Immunoblots of fresh vervet pulvinar homogenates incubated with CB1R, NAPE-PLD or FAAH antisera are shown in Figure 1A–C and demonstrate their presence across the main pulvinar subdivisions, PL + PI and PM. The specificity of the antibodies is shown by specific band recognition and was also characterized previously in the vervet monkey retina, lateral geniculate nucleus and nucleus accumbens [23,24,26]. The CB1R blot recognized the expected major band at 60 kDa and the signal was abolished when the primary antibody was incubated with its corresponding blocking peptide (Figure 1A). The NAPE-PLD immunoblot showed, as expected, an intense band at 46 kDa (Figure 1B) and the FAAH blot showed a band at 63 kDa (Figure 1C). The loading controls for each immunoblot showed levels of protein content across each condition. We provide here for the first time a set of results in the vervet monkey visual pulvinar.

### 3.2. DAB Immunohistochemistry

**Figure 2:**
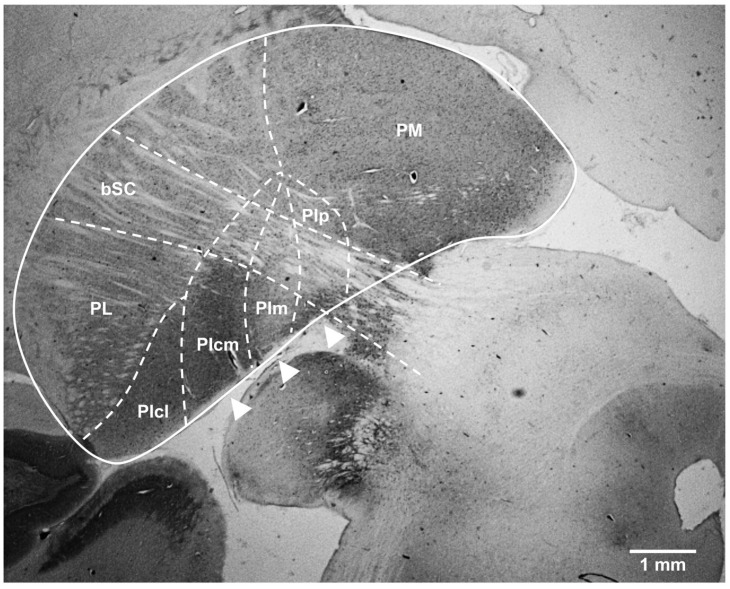
Cytoarchitectonic boundaries of the subdivisions and nuclei of the pulvinar in the vervet monkey as revealed by calbindin staining. In a coronal section of the right pulvinar (solid line) processed for calbindin-DK28, the dashed lines outline the approximate location of the anatomical borders separating the main nuclei of the pulvinar thalamic complex according to the nomenclature of Stepniewska and Kaas [5]. The three PI nuclei integrating the dorsal stream of visual processing—PIcm, PIm and PIp—are indicated by arrowheads. These nuclei rest on the ventromedial aspect of the pulvinar and are bisected by the brachium of the superior colliculus (bSC) in their upper limits. bSC: brachium of the superior colliculus; PIcl: central lateral nucleus of the inferior pulvinar; PIcm: central medial nucleus of the inferior pulvinar; PIm: medial nucleus of the inferior pulvinar; Pip: posterior nucleus of the inferior pulvinar; PL: lateral pulvinar; PM: medial pulvinar. Scale bar = 1 mm.

**Delineation of the nuclei of the inferior pulvinar.** The BrainMaps online atlas was used to define the region of interest. The pulvinar complex was localized medially and dorsally to the lateral geniculate nucleus and laterally and dorsally to the superior colliculus. The fiber bundle that forms the brachium of the superior colliculus (bSC) was clearly identifiable in all our sections and was used as an anatomical hallmark to accurately identify the PI, which is situated mostly below this structure. Hence, all our results concern the region of the PI lay ventrally to the bSC and extended from the border with the PL-PIcl nuclei laterally (discernible by its striate appearance) to the contour of the pulvinar thalamic complex medially. To better characterize this region, calbindin-D28k DAB immunostaining was performed because the immunocytochemical detection of this calcium-binding was proven to be especially informative to delimitate the nuclei within the PI subdivision across a large variety of primate species. The calbindin-immunolabeling profile observable in a coronal section of the vervet monkey brain at a low magnification (Figure 2) is in every way comparable with the pre-existent literature concerning other species of Old World and New World monkeys (e.g., [5,11]) where three functionally grouped nuclei in the PI can be identified (in addition to a more laterally located nucleus, PIcl, which is functionally more related to the PL subdivision of the pulvinar and was not the object of our study). PIcm, PIm and PIp (Figure 2, arrowheads), labeled from lateral to medial according to the nomenclature of Stepniewska and Kaas [5], exhibited different immunoreactive patterns where PIm featured as a distinct CB-depleted nucleus (sometimes referred to as the “calbindin hole”), flanked by densely CB-labeled PIcm and PIp. The confirmation of the detailed anatomy of the PI served as a reference for the presumptive localization of this collection of nuclei in subsequent image acquisitions of our immunofluorescent experiments using confocal microscopy.

### 3.3. Double Immunofluorescence

**Figure 3:**
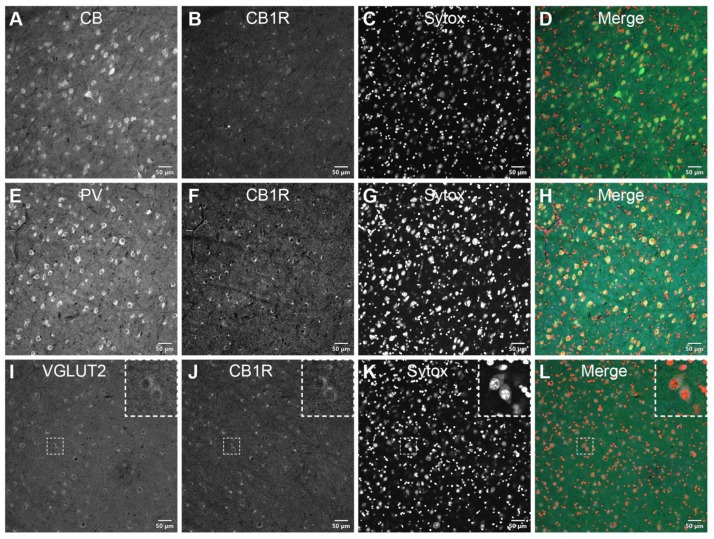
CB1R distribution in the inferior pulvinar. CB-positive (**A**–**D**) and PV-positive (**E**–**H**) neurons are shown in adjacent coronal sections of the vervet monkey pulvinar. In opposition to this diffuse and intense perinuclear staining pattern, CB1R-immunoreactivity closely matches the VGLUT2 annular-shaped terminal-like distribution (**I**–**L**, insets). The dashed squares in panels **I**–**L** indicate representative cells displayed at a higher magnification in the respective insets (3 × digital zoom). Nuclear counterstaining with Sytox orange is shown in the third column of each panel. CB: calbindin-D28k; CB1R: cannabinoid receptor type 1; PV: parvalbumin; VGLUT2: vesicular glutamate transporter 2. Scale bar = 50 µm.

**CB1R is present in VGLUT2-positive axon terminals, in the vicinity of CB+ and PV+ neurons throughout the PI.** Double immunostaining of CB1R with different cell molecular markers was carried out to examine the receptor’s expression at the cellular level. As expected, the calcium-binding proteins CB (Figure 3A–D) and PV (Figure 3E–H) were expressed in the extranuclear soma and proximal dendrites of neurons residing in the PI. Over these adjacent sections, the CB1R expression rather resembled that of VGLUT2 (Figure 3I–L) with a ring-shaped IF signal surrounding the CB+ and PV+ cell bodies. CB1R/VGLUT2 colocalization presumptively corresponded with the terminations of neurons originating in the lower superficial gray layers of the SC, normally observed in the PIcm and PIp nuclei of the PI.

**Figure 4:**
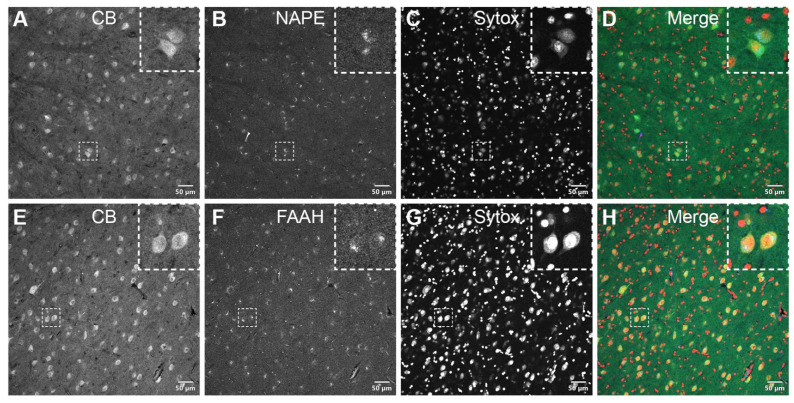
Double-label immunofluorescence illustrating the co-localization of eCB-synthesizing—NAPE-PLD (**A**–**D**, insets)—and degrading—FAAH (**E**–**H**, insets)—enzymes with calbindin in the somatodendritic compartment of PI neurons. The dashed squares in panels **A**–**H** indicate representative cells displayed at a higher magnification in the respective insets (3 × digital zoom). Nuclear counterstaining with Sytox orange is shown in the third column of each panel. CB: calbindin-D28k; FAAH: fatty acid amide hydrolase; NAPE-PLD: *N*-acyl phosphatidylethanolamine phospholipase D. Scale bar = 50 µm.

**NAPE-PLD and FAAH are expressed in CB-positive neurons within the PI.** NAPE-PLD (Figure 4A–D) and FAAH (Figure 4E–H), crucial enzymes of the eCB metabolism, were expressed in CB-positive neuronal cells of the PI. Confocal micrographs of the pulvinar co-immunolabeled for CB and NAPE-PLD or FAAH showed an overlapping localization of these markers in individual neuronal cell bodies but not within the nucleus, as seen with the nuclear stain Sytox.

## 4. Discussion

To the best of our knowledge, our study constitutes the first attempt to map the eCB system in the visual pulvinar. Considering the results of our previous study conducted in the dLGN, another nucleus of the visual thalamus, where the expression of eCB proteins was most notorious in the magnocellular layers [24], we restricted our analysis to the PI, a subdivision known to subserve the dorsal stream of visual processing. We have shown here that CB1R was expressed in the VGLUT2+ presynaptic terminals, in agreement with the ring-shaped CB1R-immunoreactivity surrounding the CB+ and PV+ cells. More precisely, CB1R/VGLUT2 colocalization hinted at the lower superficial gray layers of the SC as the likely origin of such terminations. As for the eCB-synthetizing and degrading enzymes, NAPE-PLD and FAAH, they displayed a similar expression pattern presumably corresponding with the postsynaptic somatodendritic compartment of the neurons located within the PI. By demonstrating the presence of the eCB system at this level, this work underpins the putative contribution of the eCB system to visual perception, particularly to functions assigned to the dorsal stream of visual processing.

With the progressive encephalization of primate vision, where only around 10% of retinal ganglion cells (RGCs) project to extrageniculate subcortical structures [28], the functions of the visual pulvinar remain poorly understood. In this regard, the current research framework was greatly based on the notion that the internal organization of the visual pulvinar reproduces the functional segregation of cortical visual processing into two different streams [10]. The association of the visual pulvinar with the dorsal stream (particularly with the MT complex) through the PI has been extensively studied, both anatomically and functionally.

To date, it has been demonstrated that M-type RGCs (or parasol RGCs) project to the SC with no evidence supporting the existence of similar projections originating from P-type RGCs [28,29]. The selective involvement of the magnocellular pathway advocates that the SC-PI pathway contributes differently to the dorsal and ventral pathways. Of note, while parasol RGCs are the main cell type projecting to the PI via the SC, widefield RGCs have also been identified among PI-projecting RGCs and are the sole source of direct retinal inputs to the PIm known so far [29]. Widefield RGCs comprise a morphologically diverse population of non-midget non-parasol RGCs whose function stays elusive. However, their relative abundance in the peripheral retina and several subtype-specific anatomical end electrophysiological response features point to a role in early motion processing (e.g., in direction selectivity and visual guidance of movements) [30].

The improved characterization of the pulvinar anatomical circuitry in the primate has been used to explain the behavioral manifestations exhibited by patients and animal models with lesions affecting V1. In humans, as in other primates, the bulk of retinal information is relayed through the LGN to V1 with magnocellular and parvocellular inputs exhibiting a laminar-specific distribution pattern across these structures. As both streams converge cortically into V1, lesions therein greatly impair most forms of conscious visual perception (cortical blindness). However, a few individuals retain non-conscious forms of visual perception in the affected hemifield, a phenomenon named blindsight. While the spectrum of spared visual abilities by V1-bypassing pathways is broad, motion detection and visually guided control of goal-directed movements are among the most commonly observed behaviors [31,32,33]. Once again, this conceivably denotes a lesser degree of dependence of vision for action on the geniculo-striate pathway.

In fact, much of what is presently known about the functions of the pulvinar complex derives from lesional studies. It was not until recently, however, that the pulvinar was appreciated from a developmental perspective. The organization of the PI into three distinct nuclei is a phylogenetically recent feature in primate evolution and is only observed in anthropoid primates [34]. In earlier primates, the colliculo-recipient nuclei PIp and PIcm are hardly distinguishable because the retino-recipient nuclei PIm cannot be clearly individualized. Similarly, the MT area figures as a unique region of the primate visual cortex. The developmental dynamics of the PIm-MT connectivity, shedding light on the evolution of the pulvinar complex among primates, has been reported [34]. In studies using the marmoset model, the authors reported that retinal projections to PIm were established early in development, progressively regressing into adulthood [35]. Added to the striking observation that the histological maturation of V1 and the MT area seem to concur [36], a model was proposed where MT activation through retino-PIm-MT projections drove its early maturation whereas later V1 became the dominant source of MT inputs via LGN [37]. This paradigm is compatible with the current understanding of the above-mentioned blindsight phenomenon, where generally more residual visual functions are preserved when V1 lesions occur earlier in life [13]. Accordingly, without a V1 competing stimulus, the connections between the inferior pulvinar and the extrastriate cortical areas allocated to the dorsal visual stream are deemed to subsist during the course of development and mediate residual vision [35]. MT is, hence, regarded as a pivotal cortical substrate of blindsight. Additionally, using a reverse approach, by inducing an early postnatal lesion of the PIm of the marmoset, the same team of researchers showed resulting behavioral deficits in reaching and grasping in adulthood alongside structural alterations of the parietal cortex association areas responsible for visuomotor control, once again stressing the putative developmental importance of the transient retino-PIm-MT pathway [38].

The preservation across mammals of the inferior pulvinar acting as a subcortical relay for dorsal stream processing through a retino-colliculo-pulvinar-temporal pathway [10,34] likely reflects the biological relevance of motion perception during evolution, namely to survive in predator-prey relationships. In primate phylogeny, this primitive system was gradually overtaken by an expanding occipital cortex and the respective geniculo-striate pathway that incorporate both motion and detailed vision and encompass visual awareness to a great extent. As a result, Kaas and Baldwin [10] recently conceptualized the dorsal visual stream in primates as seen today as the outcome of an increased cortical input from the primary visual cortex/V1 to MT via the LGN compared with lower mammalian species. From a developmental standpoint, as previously discussed, cumulating evidence argues for the participation of the visual pulvinar in the maturation of the extrastriate cortex [35,38]. This claim is further strengthened by the normal vision developmental trajectory in humans. Humans, as with other primates, are born with fairly good motion perception when compared with other perceptual dimensions of the visual experience [39,40]. Overall, in opposition to a cortical visual system committed to the ongoing interaction and adjustment to complex environments that underlies visual cognition, the subcortical visual system, with the PI as a relay, is rather founded on a rapid detector/first responder system for evolutionarily salient stimuli [41,42].

Analogously, the endocannabinoid system is a ubiquitous neuromodulatory system with the main components of this system identifiable in nearly all animals except for a few invertebrates [43,44] Consequently, it has been postulated that its functions should also be at least partially retained. Comparative neurobiology studies have furnished cues on the physiological roles of the eCB system that appear to be ancient and highly preserved, such as locomotion and feeding [43]. Notwithstanding, it is almost certain that over the course of evolution the eCB system acquired lineage-specific functions matching distinct brain adaptations. In primates, vision reflects a flagrant case of such functional specialization owning the wider cortical representation amid all sensory systems. On an evolutionary timescale, the eCB system predates the appearance of the earliest homologs of the pulvinar complex. It is possible that, as this structure evolved, the eCB system became implicated in regulating its functions and it is equally plausible that the eCB system contributes to neural plasticity phenomena involving subcortical visual pathways subsequent to injuries of the primary visual cortex. Indeed, altered motion perception is one the visual effects found among regular cannabis users and children prenatally exposed to cannabis [45,46]. Even if these reports require more thorough investigation, our results provide neuroanatomical correlates to support these findings.

As only male subjects were included in our study, it could constitute a potential limitation to the generalization of our results. Multiple observations from rodent models and human studies have indeed argued for the existence of a sexually dimorphic eCB system but the differences identified so far are greatly restricted to the corticolimbic system [47]. To date, no evidence of sex-related differences affecting primary sensory systems and the visual system in particular has been reported. Besides, we must also add that studies carried out by our laboratory where both sexes were included showed no apparent differences in eCB expression between male and female subjects [23].

Although it was beyond the scope of this study, a global overview of the distribution of the endocannabinoid system over the whole pulvinar complex indicated that it is also spread along the PL and the PM subdivisions (results not shown). Identical to the PI, the PL is a main visual subdivision of the pulvinar albeit that its circuitry is rather involved in the ventral stream of visual processing and its nuclei are retino-topically organized [10]. The PM integrates multisensorial information from associative frontal and temporal cortices and is thus thought to be involved in higher cognitive processes [48]. Moreover, the existence of an SC-PM-amygdala pathway has been documented and such a pathway is alleged to play a crucial role in a particular form of blindsight known as affective blindsight where the emotional content of the stimulus determines the patient’s capacity of discrimination without visual awareness [42,49]. Interestingly enough, regular cannabis use seems to interfere with emotional face recognition [50]. Perhaps, a much vaster array of visual and cognitive processes can be influenced by the presence of the eCB system in the pulvinar complex.

## Figures and Tables

**Table 1:** Primary antibodies used in this study.

Antibody	Immunogen	Source	Working Dilution	RRID
CB	Recombinant protein specific to amino terminus of human Calbindin D-28K	Cell Signaling Technology, Danvers, MA, USA	DAB 1:150	AB_2687400
CB	Calbindin D-28k purified from chicken gut	Swant, Marly, Fribourg, Switzerland	IF 1:150	AB_10000347
CB1R	Synthetic peptide corresponding with the C terminal aa 461–472 of human CB1R	Abcam, Cambridge, UK	IF 1:100, WB 1:1000	AB_447623
FAAH	Synthetic peptide corresponding with the aa 561–579 of rat FAAH	Cayman Chemical, Ann Arbor, MI, USA	IF 1:100, WB 1:1000	AB_10078701
NAPE-PLD	Synthetic peptide from human NAPE-PLD aa 159–172	Cayman Chemical, Ann Arbor, MI, USA	IF 1:100, WB 1:1000	AB_10507996
PV	Parvalbumin purified from carp muscle	Swant, Marly, Fribourg, Switzerland	IF 1:150	AB_10000343
VGLUT2	Recombinant full-length rat Vesicular Glutamate Transporter 2 (VGLUT2)	Millipore, Burlington, MA, USA	IF 1:100	AB_287552

CB: Calbindin-D28k; CB1R: cannabinoid receptor type 1; DAB: 3,3′-diaminobenzidine immunostaining; FAAH: fatty acid amide hydrolase; IF: immunofluorescence; NAPE-PLD: *N*-acyl phosphatidylethanolamine-specific phospholipase D; PV: parvalbumin; VGLUT2: vesicular glutamate transporter 2; WB: Western blot.

## Data Availability

Data supporting reported results can be requested from C.M.-F. (catarina.fernandes@umontreal.ca) and J.B. (joseph.bouskila@umontreal.ca).

## References

[1] Petersen S.E., Robinson D.L., Morris J.D. (1987). Contributions of the pulvinar to visual spatial attention. Neuropsychologia.

[2] Robinson D.L., Petersen S.E. (1992). The pulvinar and visual salience. Trends Neurosci..

[3] Villeneuve M.Y., Kupers R., Gjedde A., Ptito M., Casanova C. (2005). Pattern–motion selectivity in the human pulvinar. Neuroimage.

[4] Cortes N., de Souza B.O., Casanova C. (2020). Pulvinar Modulates Synchrony across Visual Cortical Areas. Vision.

[5] Stepniewska I., Kaas J.H. (1997). Architectonic subdivisions of the inferior pulvinar in New World and Old World monkeys. Vis. Neurosci..

[6] Itaya S.K., Van Hoesen G.W. (1983). Retinal projections to the inferior and medial pulvinar nuclei in the Old-World monkey. Brain Res..

[7] Stepniewska I., QI H.-X., Kaas J.H. (2000). Projections of the superior colliculus to subdivisions of the inferior pulvinar in New World and Old World monkeys. Vis. Neurosci..

[8] Sherman S.M., Guillery R. (1998). On the actions that one nerve cell can have on another: Distinguishing “drivers” from “modulators”. Proc. Natl. Acad. Sci. USA.

[9] Baldwin M.K., Balaram P., Kaas J.H. (2013). Projections of the superior colliculus to the pulvinar in prosimian galagos (Otolemur garnettii) and VGLUT2 staining of the visual pulvinar. J. Comp. Neurol..

[10] Kaas J.H., Baldwin M.K. (2020). The evolution of the pulvinar complex in primates and its role in the dorsal and ventral streams of cortical processing. Vision.

[11] Warner C.E., Goldshmit Y., Bourne J.A. (2010). Retinal afferents synapse with relay cells targeting the middle temporal area in the pulvinar and lateral geniculate nuclei. Front. Neuroanat..

[12] Mundinano I.-C., Kwan W.C., Bourne J.A. (2019). Retinotopic specializations of cortical and thalamic inputs to area MT. Proc. Natl. Acad. Sci. USA.

[13] Bridge H., Leopold D.A., Bourne J.A. (2016). Adaptive pulvinar circuitry supports visual cognition. Trends Cogn. Sci..

[14] Zou S., Kumar U. (2018). Cannabinoid receptors and the endocannabinoid system: Signaling and function in the central nervous system. Int. J. Mol. Sci..

[15] Howlett A.C., Mukhopadhyay S. (2000). Cellular signal transduction by anandamide and 2-arachidonoylglycerol. Chem. Phys. Lipids.

[16] Di Marzo V., Fontana A., Cadas H., Schinelli S., Cimino G., Schwartz J.-C., Piomelli D. (1994). Formation and inactivation of endogenous cannabinoid anandamide in central neurons. Nature.

[17] Egertova M., Giang D.K., Cravatt B.F., Elphick M.R. (1998). A new perspective on cannabinoid signalling: Complimentary localization of fatty acid amide hydrolase and the CB1 receptor in rat brain. Proc. R. Soc. Lond. Ser. B Biol. Sci..

[18] Wilson R.I., Nicoll R.A. (2001). Endogenous cannabinoids mediate retrograde signalling at hippocampal synapses. Nature.

[19] Ohno-Shosaku T., Maejima T., Kano M. (2001). Endogenous cannabinoids mediate retrograde signals from depolarized postsynaptic neurons to presynaptic terminals. Neuron.

[20] Heifets B.D., Castillo P.E. (2009). Endocannabinoid signaling and long-term synaptic plasticity. Annu. Rev. Physiol..

[21] Schwitzer T., Schwan R., Angioi-Duprez K., Lalanne L., Giersch A., Laprevote V. (2019). Cannabis use and human retina: The path for the study of brain synaptic transmission dysfunctions in cannabis users. Neurosci. Biobehav. Rev..

[22] Ortiz-Peregrina S., Ortiz C., Casares-López M., Jiménez J.R., Anera R.G. (2021). Effects of cannabis on visual function and self-perceived visual quality. Sci. Rep..

[23] Bouskila J., Burke M., Zabouri N., Casanova C., Ptito M., Bouchard J.-F. (2012). Expression and localization of the cannabinoid receptor type 1 and the enzyme fatty acid amide hydrolase in the retina of vervet monkeys. Neuroscience.

[24] Javadi P., Bouskila J., Bouchard J.-F., Ptito M. (2015). The endocannabinoid system within the dorsal lateral geniculate nucleus of the vervet monkey. Neuroscience.

[25] Kucera R., Bouskila J., Toutoungy M., Dow R., Palmour R., Ptito M., Bouchard J.-F. (2020). AB007. Expression and localization of CB1R, NAPE-PLD, and FAAH in the primary visual cortex of vervet monkeys. Ann. Eye Sci..

[26] Kucera R., Bouskila J., Elkrief L., Fink-Jensen A., Palmour R., Bouchard J.-F., Ptito M. (2018). Expression and localization of CB1R, NAPE-PLD, and FAAH in the vervet monkey nucleus accumbens. Sci. Rep..

[27] Burke M.W., Zangenehpour S., Ptito M. (2009). Brain banking: Making the most of your research specimens. JoVE (J. Vis. Exp.).

[28] Perry V., Cowey A. (1984). Retinal ganglion cells that project to the superior colliculus and pretectum in the macaque monkey. Neuroscience.

[29] Kwan W.C., Mundinano I.C., de Souza M.J., Lee S.C., Martin P.R., Grünert U., Bourne J.A. (2019). Unravelling the subcortical and retinal circuitry of the primate inferior pulvinar. J. Comp. Neurol..

[30] Masri R.A., Percival K.A., Koizumi A., Martin P.R., Grünert U. (2019). Survey of retinal ganglion cell morphology in marmoset. J. Comp. Neurol..

[31] Bittar R., Ptito M., Faubert J., Dumoulin S., Ptito A. (1999). Activation of the remaining hemisphere following stimulation of the blind hemifield in hemispherectomized subjects. Neuroimage.

[32] Ptito A., Leh S.E. (2007). Neural substrates of blindsight after hemispherectomy. Neuroscientist.

[33] Tran A., MacLean M.W., Hadid V., Lazzouni L., Nguyen D.K., Tremblay J., Dehaes M., Lepore F. (2019). Neuronal mechanisms of motion detection underlying blindsight assessed by functional magnetic resonance imaging (fMRI). Neuropsychologia.

[34] Baldwin M.K., Balaram P., Kaas J.H. (2017). The evolution and functions of nuclei of the visual pulvinar in primates. J. Comp. Neurol..

[35] Warner C.E., Kwan W.C., Wright D., Johnston L.A., Egan G.F., Bourne J.A. (2015). Preservation of vision by the pulvinar following early-life primary visual cortex lesions. Curr. Biol..

[36] Bourne J.A., Rosa M.G. (2006). Hierarchical development of the primate visual cortex, as revealed by neurofilament immunoreactivity: Early maturation of the middle temporal area (MT). Cereb. Cortex.

[37] Warner C.E., Kwan W.C., Bourne J.A. (2012). The early maturation of visual cortical area MT is dependent on input from the retinorecipient medial portion of the inferior pulvinar. J. Neurosci..

[38] Mundinano I.-C., Fox D.M., Kwan W.C., Vidaurre D., Teo L., Homman-Ludiye J., Goodale M.A., Leopold D.A., Bourne J.A. (2018). Transient visual pathway critical for normal development of primate grasping behavior. Proc. Natl. Acad. Sci. USA.

[39] Atkinson J., Braddick O. (2020). Visual development. Handbook of Clinical Neurology.

[40] Mason A., Braddick O., Wattam-Bell J. (2003). Motion coherence thresholds in infants—Different tasks identify at least two distinct motion systems. Vis. Res..

[41] Soares S.C., Maior R.S., Isbell L.A., Tomaz C., Nishijo H. (2017). Fast detector/first responder: Interactions between the superior colliculus-pulvinar pathway and stimuli relevant to primates. Front. Neurosci..

[42] Elorette C., Forcelli P.A., Saunders R.C., Malkova L. (2018). Colocalization of tectal inputs with amygdala-projecting neurons in the macaque pulvinar. Front. Neural Circuits.

[43] Elphick M.R. (2012). The evolution and comparative neurobiology of endocannabinoid signalling. Philos. Trans. R. Soc. B Biol. Sci..

[44] Silver R.J. (2019). The endocannabinoid system of animals. Animals.

[45] Mikulskaya E., Martin F.H. (2018). Contrast sensitivity and motion discrimination in cannabis users. Psychopharmacology.

[46] Chakraborty A., Anstice N.S., Jacobs R.J., LaGasse L.L., Lester B.M., Wouldes T.A., Thompson B. (2015). Prenatal exposure to recreational drugs affects global motion perception in preschool children. Sci. Rep..

[47] Craft R.M., Marusich J.A., Wiley J.L. (2013). Sex differences in cannabinoid pharmacology: A reflection of differences in the endocannabinoid system?. Life Sci..

[48] Homman-Ludiye J., Bourne J.A. (2019). The medial pulvinar: Function, origin and association with neurodevelopmental disorders. J. Anat..

[49] Koller K., Rafal R.D., Platt A., Mitchell N.D. (2019). Orienting toward threat: Contributions of a subcortical pathway transmitting retinal afferents to the amygdala via the superior colliculus and pulvinar. Neuropsychologia.

[50] Platt B., Kamboj S., Morgan C.J., Curran H.V. (2010). Processing dynamic facial affect in frequent cannabis-users: Evidence of deficits in the speed of identifying emotional expressions. Drug Alcohol. Depend..

